# CGE Simulation Analysis on the Labor Transfer, Agricultural Technical Progress, and Economic Development in Chongqing

**DOI:** 10.1155/2014/148479

**Published:** 2014-04-14

**Authors:** Heng Wang, Maosheng Ran

**Affiliations:** School of Economics and Business Administration, Chongqing University, Chongqing 400044, China

## Abstract

The basic structure of a CGE model dividing Mainland China into two parts, including Chongqing and rest regions, is described. Based on this CGE model, both the unilateral impact and collaborative impact of two policies, agricultural technical progress and supporting policies for improving rural labor transfer on the economic development in Chongqing, are simulated and analyzed. The results demonstrate that compared with the sum of each unilateral policy effect, the collaboration of two policies has more effective impact on facilitating the labor transfer, promoting regional economic growth, and improving income and welfare of urban and rural residents.

## 1. Introduction

The promotion of rural surplus labor transfer plays an important role in China's economic development under the dual economic system. Since China's reforming and opening up, the transfer of rural labor from agriculture to nonagricultural sectors in both East China with relatively developed economy and Midwest China with relatively underdeveloped economy will improve the labor forces allocation, increase labor productivity significantly, and facilitate the economic development [1]. However, the large-scale rural labor transfer and slow growth of labor population decrease the surplus labor resources [2]. Coastal regions in East China and some regions in West China even suffered from labor shortage. Therefore, smoothing the labor transfer channels and increasing labor supply are vital to the sustainable economic development around the whole country [3]. Among so many effecting factors of labor transfer, the agricultural technical progress can increase the agricultural productivity and lower labor demand of agriculture, thus being beneficial for increasing rural surplus labor supply, facilitating rural labor transfer, and promoting regional economic development. Therefore, the comprehensive evaluation of the effect on regional economic development caused by labor transfer and agricultural technical progress is of great significance.

Previous relevant researches mainly focused on two aspects: interaction between agricultural technical progress and labor transfer [4–6] as well as positive effect of labor transfer on economic development [7–11]. These researches have two shortages. Firstly, they did not discuss the collaborative effect of rural labor transfer and agricultural technical progress on China's economic development. Secondly, most of them applied the econometric analysis which is limited in analyzing one aspect of economic system. Only few of them adopted the computable general equilibrium (CGE) analysis that takes economic system as a whole and can depict the interaction among economic subjects. Compared with econometric analysis, the CGE analysis has better systematicness and integrality. However, previous researches using the CGE method paid few attentions on China's regional economies and the important role of agricultural technical progress in labor transfer. Therefore, a biregional CGE model of China is constructed in this paper, and a simulation analysis on the labor transfer, agricultural technical progress, and economic development in Chongqing is carried out based on this CGE model.

The rest of the paper is organized as follows. The structure of the CGE model is presented in Section 2. The simulation design and scenarios are introduced in Section 3. The simulation results are analyzed in Section 4. Based on the key parameters of labor transfer, a sensitivity analysis of CGE model is made in Section 5. Conclusions of this paper are summarized in Section 6.

## 2. Overview of the Model

### 2.1. Framework of CGE Model

**Figure 1:**
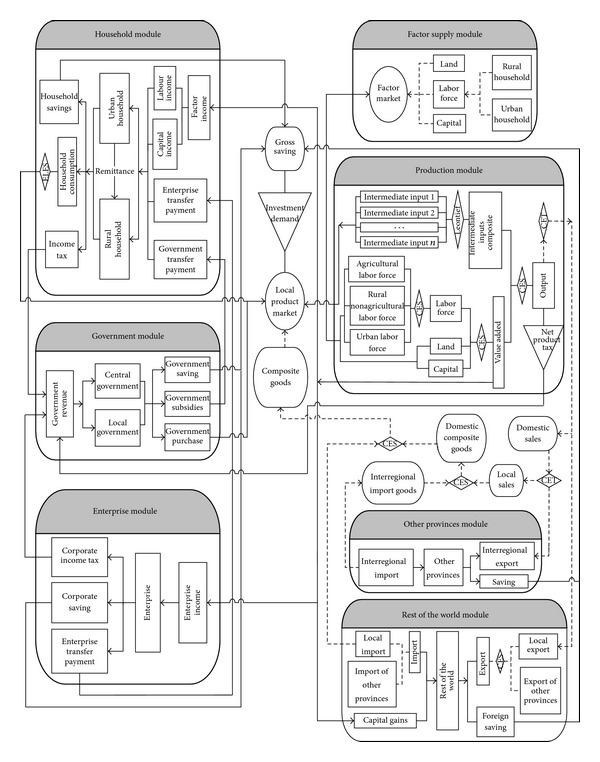
Structure of CGE model.

The CGE model used in this study divides Mainland China into two regions: Chongqing and rest of the regions in China. It involves 12 production sectors, including 1 agricultural sector and 11 nonagricultural sectors. Besides, it also involves 1 enterprise sector, 1 sector of other provinces, 1 foreign sector, 1 central government, 1 local government, 1 urban household, and 1 rural household. The production factors include labor force, capital, and land. Labor force is classified into three types: agricultural labor force, rural nonagricultural labor force, and urban labor force, according to urban and rural area as well as industry classification. Each region of China has same model structure composed of 7 modules (Figure 1). In Figure 1, the full line represents the income and expenditure flows of each model subject, while the dotted line represents the flows of production factors and products.

The production activity of the CGE model is modeled by using the multilayer nested CES (constant elasticity of substitution) function. In the first level of nest, the gross output is composed of added value and the composite of intermediate inputs. The composite of intermediate inputs is then composed of all kinds of intermediate inputs in form of a Leontief function. In the second level of nest, the added value combines the production factors land, capital, and labor force. Therein, land is only used by agricultural sector, while capital is partially mobile across local sectors and regions. In the last level of nest, labor force is composed of agricultural labor force, rural nonagricultural labor force, and urban labor force. The agricultural labor force is only used by agricultural sector, while the rest are used by nonagricultural sectors.

The product market is described in two aspects of supply and demand in this CGE model. In aspect of supply, the model follows the small country assumption in foreign trade and describes the export demand with demand curve of constant elasticity. The output of each region is allocated between domestic sales and export through the CET (constant elasticity of transformation) function. Domestic sales are allocated between local sales and interregional export through the CET function again. In aspect of demand, the total goods demand is composed of intermediate input demand, consumption demand of urban and rural residents, consumption demand of central and local governments, and investment demand. The consumption demand of urban and rural residents is depicted by the extend linear expenditure system (ELES), while the consumption demand of governments (central government and local government) and investment demand are described by fixed-share expenditure function. The CGE model applies the Armington assumption: domestic goods and imported goods are imperfect substitutes and local goods and interregional imported goods are imperfect substitutes. The total goods demand is satisfied by composite goods which are modeled by using the nested CES function. Composite goods are composed of domestic composite goods and imported goods, and then domestic composite goods are composed of local goods and interregional imported goods.

The CGE model is a recursive dynamic model and its simulation time starts from 2007 to 2015. Dynamics in the model originate from productive factors and productivity changes. The macroclosure of the model involves 3 identities which keep balance of the following 3 macroeconomic accounts: investment saving, government budget, and balance of payments. In the investment-saving balance, the total investment is determined by the available savings in the region. In the governmental budget balance, tax rates and transfers are exogenous. Government expenditure account for a fixed proportion of the government income and government saving endogenously adjusts to maintain the balance. In the balance of payments, foreign capital inflow is exogenous, while the exchange rate endogenously adjusts to maintain the balance.

### 2.2. Description of Labor Transfer in CGE Model

The CGE model makes several hypotheses concerning the labor transfer: (1) the urban labor supply is exogenous, urban labor is perfectly mobile across nonagricultural sectors within urban areas but not mobile across regions, and it cannot be transferred from urban to rural areas; (2) the rural labor supply is exogenous, rural labor is mobile across sectors and regions, it can be transferred to nonagricultural sectors in urban areas or local rural areas but cannot be transferred to rural areas of other regions; (3) the rural labor forces migrated to cities are included in urban residents (here, residents are classified according to the place of residence rather than registration alteration, and rural labor forces migrated to cities will send some of their incomes to rural residents).

Rural household is the supplier of all types of rural labor force. Following the method proposed by Hertel and Zhai [9], CET function is used to describe the labor allocation of rural household. To achieve maximum expected revenue of labor force, rural household adopts the optimal decision of labor allocation. This decision of labor allocation is expressed as (1), where the subscript *i* = 1, 2 represents the agricultural sector and nonagricultural sector, *Y* is the total labor income, *L* is the quantity of labor force, *W* is the expected labor revenue, *β* is a CET parameter, and *σ* is elasticity of transformation:

(1) max⁡ Y=∑i(Li∗Wi)s.t.  L=[∑i(βi∗Li(σ+1)/σ)]σ/(σ+1)

(2) Lagrange=∑i(Li∗Wi)+A{L−[∑i(βi∗Li(σ+1)/σ)]σ/(σ+1)}    

(3) $$\begin{array}{c} L_{i}={\left(\frac{W_{i}}{A*\beta_{i}}\right)}^{\sigma }*L \end{array}$$

(4) $$\begin{array}{c} \frac{L_{1}}{L_{2}}=\frac{\alpha_{1}}{\alpha_{2}}*{\left(\frac{W_{1}}{W_{2}}\right)}^{\sigma }. \end{array}$$

Firstly, use the Lagrange factor (*A*) to establish the Lagrange function (see (2)). Then, based on (2), the first-order partial derivative of *L*_*i*_ can be calculated. Let the first-order partial derivative of *L*_*i*_ be 0; then the optimal *L*_*i*_ can be achieved (see (3)). The labor transfer equation (see (4)) from agricultural sector to nonagricultural sector can be gained by comparing *L*_1_ and *L*_2_, where *α*_1_ = *β*_2_^*σ*^, *α*_2_ = *β*_1_^*σ*^. Extending it to regional level, the following equations can be gained:

(5) $$\begin{array}{c} \frac{{LS}_{r}^{\text{rlag}}}{{LS}_{r}^{rl}+\sum_{rg}{LS}_{r,rg}^{ul}}=\frac{\alpha_{r}^{\text{rlag}}}{\alpha_{r}^{\text{rlul}}}*{\left(\frac{{\text{WA}}_{r}}{{\text{WNA}}_{r}}\right)}^{\sigma^{ag}}, \end{array}$$

(6) Lr=[(αrrlul)−1/σag∗(LSrrl+∑rgLSr,rgul)(σag+1)/σag+(αrrlag)−1/σag∗(LSrrlag)(σag+1)/σag]σag/(σag+1),

(7) $$\begin{array}{c} {\text{WNA}}_{r}=\frac{{LS}_{r}^{rl}*W_{r}^{rl}+\sum_{rg}\left({LS}_{r,rg}^{ul}*W_{rg}^{ul}*\omega \right)}{{LS}_{r}^{rl}+\sum_{rg}{LS}_{r,rg}^{ul}}, \end{array}$$

(8) $$\begin{array}{c} {\text{WA}}_{r}=W_{r}^{\text{rlag}}+\text{bool}*\lambda_{r}*\frac{{TS}_{r}*{PT}_{r}}{L_{r}}. \end{array}$$

The subscripts *r* and *rg* represent regions and the superscripts rlag, *rl*, and *ul* represent agricultural labor force, rural nonagricultural labor force, and urban labor force, respectively; *L*_*r*_ is the initial quantity of labor force supplied by rural household; *LS*_*r*_^*l*^ is the final quantity of type l labor force provided by rural household of region *r*; *LS*_*r*,*rg*_^*ul*^ is the final quantity of labor force transferred from region *r* to region *rg*; WA_*r*_ and WNA_*r*_ are the average agricultural income and nonagricultural income of the labor force originated from rural household of region *r*; *α*_*r*_^rlag^ and *α*_*r*_^rlul^ are the CET shares of rural agricultural labor force and nonagricultural labor force originated from region *r*; *W*_*r*_^*l*^ is the average wage of type l labor force working in region *r*; *ω* is the coefficient of wage torsion which denotes the difference between the wages of transfer labor and urban labor; *TS*_*r*_ is the total supply of land; *PT*_*r*_ is the price of land; *λ*_*r*_ is the parameter for the ratio of land endowment to on-farm labor.

Equation (5) describes the rural labor transfer from agriculture to nonagricultural sectors, including nonagricultural sector in local rural areas, local urban areas, and urban areas of other regions. The quantity of labor transfer is determined by the elasticity of labor transfer (*σ* ^*ag*^) of agricultural and nonagricultural sectors as well as the relative change in average revenue of agricultural labor force and nonagricultural labor force. In the baseline scenario, *σ* ^*ag*^ is 0.6 according to the econometric research results of Sicular and Zhao [12]. The nonagricultural average revenue is defined in (7), which refers to the nonagricultural average wage of those rural labor forces transferred to nonagricultural sectors in local rural areas, local urban areas, and urban areas of other regions. The composition of average agricultural income is stated in (8), which reflects the opportunity cost for rural labor transfer from agriculture to nonagricultural sectors. In (8), bool is the policy variable of farmland market. When the CGE model involves no farmland market, rural labor transfer to nonagricultural sectors will decrease the farmland return and increase the opportunity cost of abandoning agricultural production. Under this circumstance, bool equals 1. When the CGE model has a perfect farmland market, rural labor transfers to nonagricultural sectors can lease their farmlands to others and the rent is equal to the marginal return of the farmlands. In this case, the rural labor transfer will not be influenced by farmland earnings and bool equals 0. Currently, although China has developed a farmland market preliminarily, it still has a long way to establish a unified and standard large-scale farmland market [13]. Therefore, the decision of rural labor transfer to nonagricultural sectors should take the loss of land return caused by the slow development of farmland market into account. This paper adopts the method of Zhai and Hertel [14] to determine bool = 0.5 in the baseline scenario.

Additionally, during the labor transfer, rural labor force will be attracted to urban areas due to the urban-rural wage differential. Previous researches explained the urban-rural wage differential from the perspective of labor transfer cost. Zhao [15] declared that the direct cost of rural labor transfer to urban areas (transportation, housing, and certificate transaction) accounts for 30% of the urban-rural earnings difference. Shi et al. [16] analyzed labor income differentials between urban and rural regions in China and found that there are still 48% of hourly earnings differences between urban and rural workers, which cannot be explained by labor's personal characteristics (gender, education, working experience, marriage status, and health status) and the living costs difference between urban and rural regions. This model takes the unexplained urban-rural wage differences as the indirect cost of labor transfer. During the labor transfer, urban labor and rural unagricultural labor are linked through the equilibrium condition, which is expressed by (9):

(9) $$\begin{array}{c} W_{r}^{ul}*\omega -W_{r}^{rl}={\text{CPI}}_{r}*{\text{DC}}_{r}+{\text{INDC}}_{r}, \end{array}$$

(10) $$\begin{array}{c} {\text{INDC}}_{r}={\text{TIndCost}}_{r}*W_{r}^{rl}, \end{array}$$

(11) $$\begin{array}{c} {\text{TIndCost}}_{r}=\alpha_{r}^{\text{idc}}*{\left(\frac{{LS}_{r}^{ul}}{L_{r}}\right)}^{\sigma^{\text{cmig}}}, \end{array}$$

(12) $$\begin{array}{c} {LS}_{r,rg}^{ul}=\phi_{r,rg}*{LS}_{r}^{ul}. \end{array}$$

In (9), the differential between urban wage and rural nonagricultural wage is equal to the sum of direct cost (DC_*r*_) and indirect cost (INDC_*r*_) of labor transfer. CPI_*r*_ is the consumer price index of region *r*. The CGE model uses the research results of Shi et al. [16] directly. In other words, it regards the 48% of the differential between urban wage and rural nonagricultural wage as the indirect cost of rural labor force. Let the indirect cost be the ad valorem duties of rural nonagricultural wage (TIndCost_*r*_), which is supposed as a constant elasticity function of rural labor transfer rate to urban areas. *LS*_*r*_^*ul*^ is the final quantity of labor forces transferred from rural areas of region *r* to urban areas and *α*_*r*_^idc^ is the parameter of indirect cost. During the process of rural labor transfer from rural to urban areas, the rural labor forces will choose to transfer to local urban areas or urban areas of the other region according to the income differential between the two regions. Transferred labor forces are allocated between local urban areas and urban areas of the other region through the CET function. The optimal decision is obtained from the optimal programming which has the same functions as functions (1) to (3). In the case of function (3), the share of *L*_1_ in *L* is expressed as *ϕ* and derived from the following functions:

(13) $$\begin{array}{c} \frac{L_{1}}{L}=\frac{L_{1}}{L_{1}+L_{2}}=\frac{{\left(W_{1}/\beta_{1}\right)}^{\sigma }}{{\left(W_{1}/\beta_{1}\right)}^{\sigma }+{\left(W_{2}/\beta_{2}\right)}^{\sigma }}. \end{array}$$

Multiply both sides of function (13) by *β*_1_^*σ*^∗*β*_2_^*σ*^; function (14) is obtained:

(14) $$\begin{array}{c} \frac{L_{1}}{L}*\frac{\beta_{1}^{\sigma }*\beta_{2}^{\sigma }}{\beta_{1}^{\sigma }*\beta_{2}^{\sigma }}=\frac{\beta_{2}^{\sigma }*{W_{1}}^{\sigma }}{\beta_{2}^{\sigma }*W_{1}^{\sigma }+\beta_{1}^{\sigma }*W_{2}^{\sigma }}. \end{array}$$

As mentioned earlier, *α*_1_ = *β*_2_^*σ*^, *α*_2_ = *β*_1_^*σ*^, which imply that

(15) $$\begin{array}{c} \phi =\frac{\alpha_{1}*W_{1}^{\sigma }}{\alpha_{1}*W_{1}^{\sigma }+\alpha_{2}*W_{2}^{\sigma }}. \end{array}$$

Similar to function (15), the rural labor transfer rate (*ϕ*_*r*,*rg*_) from rural areas of region *r* to urban areas of region *rg* is defined in

(16) $$\begin{array}{c} \phi_{r,rg}=\frac{\alpha_{r,rg}^{\text{mig}-ul}*{\left(W_{rg}^{ul}*\omega \right)}^{\sigma^{ul}}}{\sum_{rg}\alpha_{r,rg}^{\text{mig}-ul}*{\left(W_{rg}^{ul}*\omega \right)}^{\sigma^{ul}}}, \end{array}$$

where *α*_*r*,*rg*_^mig−*ul*^ is CET parameter and *σ* ^*ul*^ is CET elasticity.

### 2.3. Data Base and Parameters of the CGE Model

The data base of the CGE model is the social accounting matrix (SAM) of Chongqing in 2007 and SAM of other domestic regions in 2007. Considering data availability, this paper compiled the national SAM and SAM of Chongqing in 2007 firstly on the basis of China's and Chongqing's input-output table in 2007. Next, the national SAM minus SAM of Chongqing is the SAM of other domestic regions. The CGE involves two types of parameters: one mainly includes all kinds of elasticities of the model, which is determined from literature review and data exogenesis, mainly cited from the research results of Zhai and Hertel [17] and Dirk [18] as well as the data of China in the GTAP model; the other type is calibrated by the data of former type and SAM.

## 3. Simulation Scenarios Design 

The simulation scenario in this paper includes the baseline scenario and three policy scenarios. In the policy scenarios, the supporting policies for improving rural labor transfer is expressed by lowering labor transfer cost and perfecting the farmland market, meanwhile the agricultural technical progress is reflected by the growth rate of agricultural total factor productivity (TFP). Details of these scenarios are stated as below.

S0: this is the baseline scenario, in which each region has exogenous GDP growth rate and endogenous TFP growth rate. Besides, it supposes that all industries have the same TFP growth rate. The GDP growth rate from 2007 to 2010 is the practical GDP growth rate. From 2011 to 2015, the annual average GDP growth rate of Chongqing is 12.5% and the annual average GDP growth rate of other domestic regions is 7.8% (the annual average GDP growth rate of Chongqing from 2011 to 2015 is set according to its “12th Five-year Plan.” Meanwhile, Li Shantong et al. (2009) determined China's annual average GDP growth rate from 2011 to 2015 as 7.9% in the “prospect of China's economic growth from “12th Five-year Plan” to 2030”. Based on the above two data, this paper estimated the annual average GDP growth rate of other domestic regions from 2011 to 2015 as 7.8%). In each region, the growth rate of labor and population, as well as the farmland supply, are exogenous. In addition, the total current capital stock of China is the sum of previous capital stock deducting capital depreciation and the gross investment of China.
S1: this is the scenario of improvement of supporting policies to labor transfer. The cost of rural labor transfer includes transportation cost, living cost, psychological cost, job training cost, and opportunity cost caused by unemployment during the transfer [19]. The CGE model supposes that government adopts various measures to lower the transfer cost by 5% by 2015 relative to 2007 levels, such as providing skill training and accommodation supports, removing institutional obstacles of rural labor transfer, and offering social insurance. In addition, the model also supposes that government facilitates the revolution of farmland transfer and establishes a perfect farmland market to let bool = 0.
S2: this is the scenario of agricultural technical progress. It supposes that the annual TFP growth rate of agriculture in S2 is 0.1% higher than that in S0.
S3: this is the combination of S1 and S2. In other words, the rural labor transfer cost will be lowered by 5% by 2015 relative to 2007 levels, bool is equal to 0, and the annual TFP growth rate of agriculture in S3 is 0.1% higher than that in S0.

## 4. Simulation Results

The above four scenarios are simulated in this paper by using the CGE model. Next, the simulation results of S1, S2, and S3 are compared with those of S0. The comparison results are the final simulation results, which cover the following three aspects.

### 4.1. Impact on Labor Transfer and Wage

The labor transfer and wage change of all labor forces in 2015 are presented in Table 1. Compared with S0, the rest of the scenarios (S1, S2, and S3) witness a growth of labor transfer from agriculture to nonagricultural sectors and from rural areas to urban areas. The labor transfer in S3 is higher than the sum of that in S1 and S2. Additionally, all labor forces are enjoying increasing wages in three scenarios except for the average urban wage in S1 and S3 as well as the average agricultural wage and the average rural wage in S2. The influence of average agricultural wage on average rural wage is greater than that of rural nonagricultural wage, and the average rural wage has the similar variation tendency to the average agricultural wage. Viewed from numbers, the average agricultural wage in S3 ends the decrease in S2 and increases slightly. The average urban wage in S3 decreases less than that in S1. These indicate that, on one hand, the policy effect in S3 is not a simple superposition of policies in S1 and S2 but is more effective in facilitating the labor transfer than the sum of S1 and S2 due to interaction between S1 and S2. On the other hand, S1 decreases the quantity of the rural labor force and increases the urban labor supply; S2 not only decreases the agricultural labor demand but also drives the development of nonagricultural sectors through the industrial linkages and increases the labor demand of nonagricultural sectors. Since the macroclosure of this CGE model has a hypothesis of full employment, the average agricultural wage in S1 and the average urban wage in S2 are raised, while the average urban wage in S1 and the average agricultural wage in S2 are negatively affected. S3 is a composite scenario made up of S1 and S2. Under the interaction of S1 and S2, the negative impact on average urban wage in S3 is smaller than that in S1; the negative impact in S2 on average agricultural wage is offset, and the average agricultural wage even increases slightly in S3.

### 4.2. Impact on Regional Macroeconomic Variables

The variation of macroeconomic indexes of Chongqing in three policy scenarios is listed in Table 2. Among these, the residents' consumption variation is determined by the variation of urban and rural residents' consumptions. According to the simulation results, the impact on urban and rural residents' consumption depends on two factors. On one hand, policy change will attract rural labor forces from rural areas to urban areas and change the population of two kinds of residents (decrease the rural population and increase the urban population), thus decreasing the rural residents' consumption and increasing the urban residents' consumption. On the other hand, rural labor transfer results in wage change (Table 1). Increase of average rural wage will increase the income of rural residents, thus increasing the rural residents' consumption. Similarly, decrease of average urban wage will lower the income of urban residents, thus decreasing the urban residents' consumption. Results demonstrate that all the three policy scenarios decrease rural residents' consumption and increase urban residents' consumption, finally increasing the residents' consumption. Furthermore, the investment, real GDP, and GDP per capita of Chongqing in all scenarios are increased except the investment in S1. It can be seen from Table 2 that the growth of a variable (residents' consumption, investment, real GDP, and GDP per capita) in S3 is greater than the sum of that in S1 and S2. Therefore, attentions shall be paid to the collaborative effect of S1 and S2 during the boost of regional economic growth.

### 4.3. Impact on Urban and Rural Residents

The variation of per capita incomes and welfares of urban and rural residents in all three scenarios is listed in Table 3. The variation of welfares is measured by equivalent variation (EV).

S1 increases the per capita incomes and welfares of rural residents but decreases that of urban residents. S2 is in favor of urban residents but decreases the per capita incomes and welfares of rural residents. Compared with S1, S3 decreases the per capita incomes and welfares of urban residents more slowly. Unlike S2, S3 has a positive effect on the per capita incomes and welfares of rural residents. This indicates that S3 is superior to S2 and S1, which can not only avoid negative impact of S2 on rural residents but also relieve the negative impact of S1 on urban residents.

## 5. Sensitivity Analysis

Based on the key parameters of labor transfer, a sensitivity analysis is made to ensure the reliability of the results. In sensitivity analysis, the elasticity of labor transfer is 2.2 (according to the econometric research results of Sicular and Zhao, for the decrease of the agricultural wage, the elasticity of labor transfer is 2.2), and the simulations of the above four scenarios are repeated by CGE model. The comparison results of S1, S2, and S3 relative to S0 are presented in Table 4.

It can be seen by comparing the results in Table 4 to the previously simulation results (Tables 1–3), the quantities of labor transfer from agriculture to nonagricultural sectors and from rural to urban areas are increased in the same scenario. In S2, the negative impact of the reduction of agricultural labor demand on average agricultural wage is relieved, so the decreasing rate of the average agricultural wage and average rural wage as well as the increasing rate of the average urban wage are getting lower. The per capita income of rural residents in S2 is no longer reduced, but with a slight rise. And the growth of per capita incomes of urban residents is also reduced. The increasing of transfer labor force leads to an increase of urban population and decrease of rural population. In S1 and S3, the average agricultural wage, the average rural wage, and the per capita incomes of rural residents are subject to the more positive impact, while the average urban wage and the per capita incomes of urban residents are subject to the greater negative impact. Therefore, under the dual action of changes in the income and population of residents, the increasing rate of urban residents' consumption and decreasing rate of rural residents' consumption were reduced, therefore leading to a decrease of residents' consumption in S1, as well as the increasing rate of residents' consumption in S2 and S3. In addition, increasing the transfer labor simultaneously enlarges the negative impact of S1 on investment, as well as the positive impact of S2 on investment. The increasing rate of investment in S3 is lower because of the collaboration of S1 and S2. In addition to the above variables, the positive and negative effects that other variables were subject to are amplified, but the change contrary to theoretical expectations does not exist. In a word, all kinds of variables are in accordance with theoretical expectations, and the basic conclusion the paper derived from CGE model is robust.

## 6. Conclusion

This paper establishes a biregional CGE model of China. It designs three policy scenarios based on the supporting policies for improving labor transfer and agricultural technical progress. Finally, it carries out a simulation analysis and a sensitivity analysis on the impact of these policy scenarios on the economic development of Chongqing, finding that the collaborative effect of supporting policies for improving labor transfer and agricultural technical progress is superior to their unilateral effect in boosting the economic development of Chongqing. Such superiority is mainly manifested in three aspects: (1) the combination of supporting policies for improving labor transfer and agricultural technical progress can facilitate the surplus rural labor transfer in Chongqing positively. Compared with the sum of unilateral policy effect, it intensifies the labor transfer and increases the quantity of labor transfer. Secondly, except for increasing labor wage, it also relieves the negative impact of agricultural technical progress on average agricultural wage and average rural wage as well as the negative impact of labor transfer promotion on the average urban wage. (2) The combination of supporting policies for improving labor transfer and agricultural technical progress can boost the economic growth in Chongqing. It not only drives the growth of residents' consumption, practical GDP, and GDP per capita in Chongqing but also relieves the negative impact of labor transfer on investment. (3) The combination of supporting policies for improving labor transfer and agricultural technical progress can improve the incomes and welfares of urban and rural residents. During the labor transfer, it not only increases the per capita income and welfare of rural residents and avoids negative impact of agricultural technical progress on rural residents, but also offsets the negative impact of labor transfer on urban residents.

## Figures and Tables

**Table 1:** Labor transfer and wages in 2015 (% change).

	S1	S2	S3
Labor transfer			
Off-farm labor	0.4268	0.1398	0.5691
Rural—urban	38.5546	1.6546	40.7197
Wages			
Agricultural	0.4255	−0.4086	0.0171
Rural nonagri.	0.3321	0.1554	0.4932
Rural	0.3331	−0.1505	0.1833
Urban	−0.2460	0.1330	−0.1162

Data source: simulation results.

**Table 2:** Macroeconomic variables in 2015 (% change).

	S1	S2	S3
Regional real GDP	0.0852	0.0457	0.1318
Regional per capita GDP	0.2037	0.0500	0.2555
Residents' consumption	0.0146	0.1346	0.1497
Urban residents	−0.3653	−0.1545	−0.5266
Rural residents	0.0733	0.1793	0.2543
Investment	−0.0088	0.2238	0.2175

Data source: simulation results.

**Table 3:** Urban and rural residents per capita income and welfare (EV) in 2015 (% change).

	S1	S2	S3
Per capita income			
Rural residents	1.0912	−0.0787	1.0243
Urban residents	−0.3385	0.1937	−0.1504
*Per capita EV			
Rural residents	0.0164	−0.0030	0.0135
Urban residents	−0.0103	0.0029	−0.0075

Data source: simulation results. (*Change of original value.)

**Table 4:** Results of policy simulations in 2015 (% change, *σ* ^ag^ = 2.67).

	S1	S2	S3
Labor transfer			
Off-farm labor	0.7771	0.2489	1.0325
Rural—urban	42.4364	1.8939	45.0041
Wages			
Agricultural	0.6921	−0.1481	0.5476
Rural nonagri.	0.1423	0.0722	0.2183
Rural	0.4712	−0.0193	0.4545
Urban	−0.3023	0.0866	−0.2201
Regional real GDP	0.1121	0.0668	0.1803
Regional per capita GDP	0.2528	0.0710	0.3263
Residents' consumption	−0.0012	0.1295	0.1288
Rural residents	−0.3462	−0.0983	−0.4532
Urban residents	0.0535	0.1656	0.2211
Investment	−0.0758	0.2421	0.1696
Per capita income			
Rural residents	1.2763	0.0080	1.3016
Urban residents	−0.3880	0.1778	−0.2176
*Welfare (EV)			
Rural residents	0.0198	−0.0011	0.0190
Urban residents	−0.0113	0.0030	−0.0086

Data source: simulation results. (*Change of original value.)
